# Gender differences in acute recreational drug toxicity: a case series from Oslo, Norway

**DOI:** 10.1186/s12873-019-0244-3

**Published:** 2019-04-29

**Authors:** Victoria Lykke Syse, Mette Brekke, Marit Mæhle Grimsrud, Per Sverre Persett, Fridtjof Heyerdahl, Knut Erik Hovda, Odd Martin Vallersnes

**Affiliations:** 1 Faculty of Medicine, University of Oslo, Oslo, Norway; 2 Oslo Accident and Emergency Outpatient Clinic, Department of Emergency General Practice, City of Oslo Health Agency, Oslo, Norway; 3 General Practice Research Unit, University of Oslo, Oslo, Norway; 4 The Norwegian PSC Research Center, Oslo University Hospital, Oslo, Norway; 5 Department of Acute Medicine, Oslo University Hospital, Oslo, Norway; 6 Department of Prehospital Medicine, Oslo University Hospital, Oslo, Norway; 7 Norwegian Air Ambulance Foundation, Oslo, Norway; 8 The Norwegian CBRNe Centre of Medicine, Department of Acute Medicine, Oslo University Hospital, Oslo, Norway; 9 Department of General Practice, University of Oslo, Oslo, Norway

**Keywords:** Poisoning, Intoxication, Recreational drugs, Gender differences

## Abstract

**Background:**

Female drug users report poorer physical and mental health than male drug users. We describe female and male patients treated for acute recreational drug toxicity, and look for gender differences in clinical state, treatment, and toxic agents taken.

**Methods:**

Retrospective case series from a primary care emergency outpatient clinic and a hospital emergency department in Oslo, Norway. All patients treated for acute recreational drug toxicity from October 2013 through March 2015 were included, except patients with lone alcohol intoxication. Patients were grouped according to whether they had taken opioids or not, as a proxy differentiation between heavy drug users and party drug users. Data from the two clinical settings were analysed separately.

**Results:**

In total, 2495 cases were included, 567 (22.7%) were women. Female patients were younger than males, median 31 vs 34 years (*p* < 0.001). On most comparisons of clinical variables there were no significant differences between genders. A larger proportion of females in the outpatient opioid group were hypotensive, 10.9% vs 3.9% (*p* < 0.001). Fewer females were intubated, none vs 21.1% (*p* = 0.019) in the hospital opioid group, and 6.4% vs 21.0% (*p* = 0.039) in the hospital non-opioid group. The proportion of gamma-hydroxybutyrate (GHB) poisoning was larger among females both at the outpatient clinic (14.4% vs 8.6%, *p* < 0.001) and at the hospital (60.3% vs 36.4%, *p* = 0.001), while the proportion of heroin poisoning was smaller among females at the outpatient clinic (37.1% vs 47.0%, *p* < 0.001).

**Conclusion:**

One in four patients treated for acute recreational drug toxicity were women. Female patients were younger, had more frequently taken GHB and were less frequently intubated. Otherwise, the gender differences regarding clinical state and treatment were small. Although female drug users are known to report poorer health than males, we did not find that women had a more severe clinical course than men when presenting with overdose.

## Background

Recreational drug use is widespread. More than one in four European adults have used a recreational drug other than alcohol at some time, most commonly cannabis [1]. This is also the case in Norway [2]. Apart from alcohol and cannabis, the most widely used recreational drugs in Norway are cocaine and amphetamines, with life-time prevalences of use at about 5 %, methylenedioxymethamphetamine (MDMA) at about 2 %, and gamma-hydroxybutyrate (GHB), lysergic acid diethylamide (LSD) and heroin at about 1 % [2, 3]. The lifetime prevalence of use of illegal drugs is two to three times as high among men as among women in European population surveys [1, 2]. The same pattern is found in Norway, also when looking at specific drugs [2, 3]. Furthermore, the proportion of females among patients entering treatment for drug use varies from 14 to 28% for the major illegal drugs in Europe [1]. In Norway, one in three patients in opioid substitution treatment are females [2], as are one in three Norwegian high-risk drug users [2, 4, 5]. Among the high-risk users, mainly using opioids, about 80% of both males and females inject their drugs [2, 5].

The male preponderance in the prevalence of drug use corresponds well both to a recent European multi-centre study and to previous Norwegian studies on patients treated for acute recreational drug toxicity, in which one in four were females [6–8]. In the Norwegian studies, when looking at specific drugs, the proportion of females were in the range of 20–30% [7, 8]. Gender distribution for individual drugs was not detailed in the European study [6]. Gender differences in clinical features were not reported in any of these studies. Studies of emergency department presentations for acute recreational drug toxicity are sparse [9], and to the best of our knowledge, gender differences in the clinical course and treatment of acute recreational drug toxicity have not been studied.

Apart from the larger number of males ever having used a recreational drug other than alcohol, gender differences in health among party drug users have not been reported [10, 11]. Subjective effects of recreational drugs are reported similarly by men and women, except that women report less effect of amphetamines and cocaine when in the luteal phase of the menstrual cycle [12].

On the other hand, the general health of female heavy drug users seems to be poorer compared to their male counterparts. In a US cohort of individuals in their fifties with a history of heroin dependence, women reported more chronic health problems and more psychological distress than men, and poorer health and functioning than the general female population of comparable age [13]. The poorest mental health was reported among those still using drugs or alcohol [13]. Similar findings have been reported among opioid users enrolling in a US detoxification program [14]. An Australian study among patients in an opioid replacement treatment program found women more likely to have a history of maltreatment as children, to experience violence and sexual exploitation as adults, and to have a higher prevalence of psychiatric disorders [15]. In a multi-centre Spanish study, young female opiate users reported poorer health related quality of life than males [16]. In Norway, one in four drug overdose deaths are among women [2]. Though males with substance use disorders have a higher absolute risk of premature death than females, standardised mortality rates are higher among female drug users, indicating a larger increase in relative risk of premature death associated with substance use disorders among females [17]. As far as we know, it has not been studied whether the reported poorer physical and mental health among female drug users result in a more severe clinical course when presenting to emergency medical services with acute recreational drug intoxication.

Gender differences in the clinical presentation of acute recreational drug toxicity may have implications for patient management. Emergency medical personnel might have to be alert to different signs and symptoms in females than in males when assessing the severity of the toxicity.

### Aims

We look for gender differences in patients treated for acute recreational drug toxicity, by comparing female and male patients as to toxic agents taken, clinical state, and treatment.

## Methods

### Study design

Retrospective case series from a primary care emergency outpatient clinic (the Oslo Acute and Emergency Outpatient Clinic (OAEOC)) and a hospital emergency department (Oslo University Hospital (OUH)). We used the case definition and variable set developed by the European Drug Emergencies Network [18].

### Settings

The emergency medicine service in Norway is two-tiered. Patients cannot present at hospital emergency departments directly, but must initially be seen by a doctor in primary care or by the ambulance service. In addition to the service delivered by general practitioners (GPs) during office hours, primary care emergency services are run by GPs on rotation after office hours or by larger primary care emergency outpatient clinics.

The OAEOC is the main primary care emergency outpatient clinic in Oslo, serving the entire city at all hours. Diagnostic resources and treatment options are limited, but there are facilities for short-time observation. The majority of patients with acute recreational drug toxicity in Oslo are treated at the OAEOC, though the more severely poisoned patients are brought directly to hospital by the ambulance service [7]. Patients presenting to the OAEOC in need of diagnostic procedures or treatment beyond what is locally available, are transferred to hospital. OUH is one of four hospitals in Oslo treating patients with acute poisoning, with a catchment area encompassing about one third of the city’s population. Oslo had a population of 647,676 as per 1 January 2015 [19].

### Participants

We included all patients presenting to the OAEOC or OUH from October 2013 through March 2015 (18 months) with symptoms and/or signs consistent with acute recreational drug toxicity and/or directly related to recreational drug use. Recreational drug use was defined as the use of any psychoactive compound for recreational purposes, encompassing classic substances of abuse, new psychoactive substances, plants, fungi, herbal medicines, industrial and/or domestic products, and licensed pharmaceutical preparations. Patients with alcohol as the only toxic agent were excluded. Furthermore, we excluded patients who had taken drugs to self-harm or as a suicide attempt, and patients with poisoning inflicted by others, as spiked drinks or date-rape drugs. Patients with additional complaints were included if the recreational drug toxicity in itself required observation and/or treatment.

Eligible cases were identified retrospectively from the patient registration lists in the local electronic medical records. Inclusion was based on the information in the case history as noted in the electronic medical records by the doctor treating the patient.

### Data collection and classification

Data was collected from the local electronic medical records at both locations, and at the OAEOC also from local observation charts.

We registered gender, age, toxic agents taken, whether the patient was brought by ambulance, length of stay, treatment given, disposition from the outpatient clinic or hospital emergency department (admitted critical care unit, admitted psychiatric ward, admitted other hospital unit, medically discharged, or self-discharge), and whether the patient died. Furthermore, we registered vital signs on arrival (respiratory rate, heart rate, blood pressure, temperature, and conscious level by Glasgow Coma Scale) and clinical features of the toxic episode (vomiting, hyperthermia, headache, anxiety, hallucinations, agitation, psychosis, seizures, cerebellar features, palpitations, chest pain, hypertension, hypotension and arrhythmias). At the hospital, we also registered blood lactate levels on arrival, and peak serum levels of creatine kinase (CK) and creatinine. The latter two were only registered during the first year of the data collection. None of these blood tests are done at the OAOEC.

Toxic agents were registered as assessed and noted in the electronic medical records by the doctor treating the patient, mainly based on clinical examination and information from the patient, companions, ambulance personnel or the police. Toxicological analyses are not done at the OAEOC, while at the OUH analytical confirmation was available in 33.2% (92/277) of the cases, by means of gas chromatography – mass spectrometry (GC-MS) for GHB, and enzymatic screening for other agents followed by liquid chromatography – mass spectrometry (LC-MS) confirmation. We co-categorised methamphetamine as amphetamine and Z-hypnotics as benzodiazepines.

Treatment was defined as any intervention apart from mere observation. In addition, we specifically recorded intubation, and treatment with naloxone, flumazenil, and sedative agents.

Clinical features were registered as assessed by the doctor treating the patient. If a specific clinical feature was not recorded in the electronic medical records, it was registered as not present.

### Statistical analyses

We compared female cases and male cases on toxic agents taken, age, treatment given, length of stay, disposition, and clinical signs and symptoms.

As hospital patients systematically are more severely sick than OAEOC patients, having been selected for hospital treatment by the ambulance service or a primary care doctor, we did separate comparisons for the two locations. Among the included patients transferred to hospital from the OAEOC, 72/427 (16.9%) were sent to the OUH. As we analysed the data from the two locations separately, these 72 cases were included in the analysis at both locations.

Heavy drug users and party drug users are to some extent different populations. Heavy drug users are more likely to suffer from problems and diseases related to addiction and more extensive drug use. To avoid this possible confounding factor, we wanted to differentiate between these two groups. In Norway, injecting is the hallmark of heavy drug use, and the drugs most frequently used for injection are opioids [2, 4, 5]. As a proxy differentiation between heavy drug users and party drug users, cases with and without any opioid among the toxic agents taken were analysed separately.

Statistical analyses were done in IBM SPSS-version 25. To compare proportions, Pearson’s chi-square test was used. As our continuous variables did not follow the normal distribution, we used medians and interquartile ranges for descriptions and Mann-Whitney U-test for comparisons. When generating categoric variables from the continuous data on vital signs, missing values were treated as the relevant clinical feature not being present. Apart from this, missing values were kept out of the analyses.

### Ethics

The study was done as a quality improvement study. It was approved by the Oslo University Hospital Information Security and Privacy Office, and by the director of the Department of Emergency General Practice at the City of Oslo Health Agency.

## Results

In total, 2218 cases were included at the outpatient clinic and 277 at the hospital. There were 499/2218 (22.5%) female cases at the outpatient clinic and 68/277 (24.5%) at the hospital. Median age was 34 years (interquartile range (IQR) 26–45) at the outpatient clinic and 31 years (IQR 24–39) among the hospital patients.

The proportion of cases with GHB was larger among females, both at the outpatient clinic, 14.4% (72/499) vs 8.6% (147/1719) (*p* < 0.001), and at the hospital, 60.3% (41/68) vs 36.4% (76/209) (*p* = 0.001), though absolute numbers were larger among males (Table 1). Among females at the outpatient clinic, the proportion of cases with heroin was smaller, 37.1% (185/499) vs 47.0% (808/1719) (*p* < 0.001).

**Table 1:** Toxic agents reported in recreational drug toxicity

	Outpatient clinic		Hospital ED	
	Females *n* (%)	Males *n* (%)	Females *n* (%)	Males *n* (%)
Heroin	185 (37.1)***	808 (47.0)	19 (27.9)	59 (28.2)
Benzodiazepines	167 (33.5)	545 (31.7)	9 (13.2)	38 (18.2)
Amphetamine	120 (24.0)*	339 (19.7)	15 (22.1)	64 (30.6)
GHB	72 (14.4)***	147 (8.6)	41 (60.3)**	76 (36.4)
Cannabis	55 (11.0)	206 (12.0)	8 (11.8)	25 (12.0)
Cocaine	22 (4.4)	115 (6.7)	5 (7.4)	34 (16.3)
MDMA	16 (3.2)	45 (2.6)	3 (4.4)	7 (3.3)
Methadone	12 (2.4)	48 (2.8)	–	2 (1.0)
Buprenorphine	12 (2.4)	41 (2.4)	–	3 (1.4)
Other/unknown opioid	32 (6.4)	121 (7.0)	3 (4.4)	10 (4.8)
Other/unknown	33 (6.6)	116 (6.7)	12 (17.6)	35 (16.7)
Alcohol^a^	115 (23.0)	451 (26.2)	30 (44.1)	80 (38.3)
Total^b^	499 (100)	1719 (100)	68 (100)	209 (100)

^a^Patients with lone alcohol intoxication were not included, so only recorded when taken in addition to other recreational drugs

^b^As more than one drug (excluding alcohol) were taken in 38.5% (853/2218) of outpatient cases and 49.5% (137/277) of hospital cases, numbers and percentages add up to more than the total. There were no statistically significant gender differences in having taken more than one drug

ED: emergency department; GHB: gamma-hydroxybutyrate; MDMA: methylenedioxymethamphetamine

*p < 0.05, **p < 0.01, ***p < 0.001 for comparisons between genders

For the rest of the analyses we grouped the cases at the two locations according to whether they had taken opioids or not (Tables 2 and 3). At the outpatient clinic, an opioid was among the toxic agents taken in 54.8% (1215/2218) of the cases. At the hospital, an opioid was among the toxic agents taken in 33.2% (92/277) of the cases. In the opioid group, there were 18.8% (229/1215) female cases at the outpatient clinic and 22.8% (21/92) at the hospital, while in the non-opioid group there were 26.9% (270/1003) females at the outpatient clinic and 25.4% (47/185) at the hospital.

**Table 2:** Age, treatment, and disposition of patients with acute recreational drug toxicity

	Opioid among toxic agents				No opioid among toxic agents			
	Outpatient clinic		Hospital ED		Outpatient clinic		Hospital ED	
	Females *n* (%)	Males *n* (%)	Females *n* (%)	Males *n* (%)	Females *n* (%)	Males *n* (%)	Females *n* (%)	Males *n* (%)
Age^a,b^	36 (25–44)***	38 (30–46)	29 (23–31)*	35 (27–41)	29 (23–38)*	31 (25–42)	30.5 (23–35)	31.5 (24–38)
Brought by ambulance	141 (62.4)	613 (62.7)	21 (100)	66 (93.0)	122 (45.7)	356 (49.2)	44 (93.6)	129 (93.5)
Treatment required^c^	86 (37.6)*	292 (29.6)	15 (71.4)	54 (76.1)	21 (7.8)	90 (12.3)	31 (66.0)	94 (68.1)
Intubated	–	–	-*	15 (21.1)^f^	–	1 (0.1)^g^	3 (6.4)*^g^	29 (21.0)^g^
Naloxone	71 (31.0)	266 (27.0)	14 (66.7)	43 (60.6)	7 (2.6)	24 (3.3)	11 (23.4)	44 (31.9)
Flumazenil	1 (0.4)	3 (0.3)	6 (28.6)	23 (32.4)	–	–	11 (23.4)	32 (23.2)
Sedation	3 (1.3)	6 (0.6)	-*	15 (21.1)	6 (2.2)	34 (4.6)	12 (25.5)	42 (30.4)
Length of stay^a^	4:36 (2:36–6:04)	4:44 (2:45–6:31)	14:56* (4:17–33:45)	21:25 (11:14–51:06)	2:30 (1:21–4:37)	2:44 (1:24–4:40)	9:11 (5:13–20:45)	12:24 (6:17–27:37)
Disposition								
*Admitted critical care unit*	–	–	18 (85.7)	68 (95.8)	–	–	40 (85.1)	117 (84.8)
*Admitted psychiatric ward*	3 (1.3)	6 (0.6)	1 (4.8)	–	18 (6.7)	65 (8.9)	1 (2.1)	1 (0.7)
*Admitted other*^*d*^	44 (19.2)	170 (17.2)	1 (4.8)	–	61 (22.6)	152 (20.7)	2 (4.3)	11 (8.0)
*Medically discharged*	143 (62.4)	638 (64.7)	–	–	138 (51.1)	383 (52.3)	2 (4.3)	2 (1.4)
*Self-discharge*	39 (17.0)	171 (17.3)	1 (4.8)	3 (4.2)	53 (19.6)	133 (18.1)	2 (4.3)	7 (5.1)
Total	229 (100)	986 (100)^e^	21 (100)	71 (100)	270 (100)	733 (100)	47 (100)	138 (100)

Percentages refer to total at the bottom of the columns

^a^Median (interquartile range)

^b^Missing: 16 in outpatient opioid group (females: *n* = 226; males: *n* = 973), 2 in hospital opioid group (males: *n* = 69), 23 in outpatient non-opioid group (females: *n* = 265; males: *n* = 715), 5 in hospital non-opioid group (females: *n* = 46; males: *n* = 134)

^c^Any kind of treatment other than mere observation

^d^From outpatient clinic: transferred to hospital emergency department. From hospital ED: admitted to hospital department other than critical care unit or psychiatric ward

^e^One patient with unknown disposition

^f^In 13 cases also sedated

^g^All were also sedated

ED: emergency department

*p < 0.05, **p < 0.01, ***p < 0.001 for comparisons between genders

**Table 3:** Clinical features in patients with acute recreational drug toxicity

	Opioid among toxic agents				No opioid among toxic agents			
	Outpatient clinic		Hospital ED		Outpatient clinic		Hospital ED	
	Females *n* (%)	Males *n* (%)	Females *n* (%)	Males *n* (%)	Females *n* (%)	Males *n* (%)	Females *n* (%)	Males *n* (%)
Cardiac arrest^a^	–	–	1 (4.8)	7 (9.9)	–	–	1 (2.1)	1 (0.7)
Tachypnoea (RR > 20)^a^	8 (3.5)	49 (5.0)	5 (23.8)	9 (12.7)	21 (7.8)	70 (9.5)	8 (17.0)	23 (16.7)
Bradypnoea (RR < 10)^a^	42 (18.3)	144 (14.6)	2 (9.5)	10 (14.1)	2 (0.7)	10 (1.4)	3 (6.4)	4 (2.9)
Tachycardia (HR > 99)^a^	41 (17.9)	181 (18.4)	3 (14.3)	20 (28.2)	88 (32.6)	251 (34.2)	11 (23.4)	41 (29.7)
Bradycardia (HR < 50)^a^	4 (1.7)	28 (2.8)	3 (14.3)	6 (8.5)	1 (0.4)	8 (1.1)	2 (4.3)	12 (8.7)
Hypertension (SBP ≥ 180)	2 (0.9)	5 (0.5)	2 (9.5)	2 (2.8)	1 (0.4)	5 (0.7)	5 (10.6)	14 (10.1)
Hypotension (SBP ≤ 90)	25 (10.9)***	38 (3.9)	3 (14.3)	9 (12.7)	9 (3.3)	19 (2.6)	13 (27.7)	19 (13.8)
Hyperthermia (tp ≥ 39.0 °C)	1 (0.4)	10 (1.0)	1 (4.8)	7 (9.9)	3 (1.1)	5 (0.7)	2 (4.3)	3 (2.2)
GCS score^a,b^								
*15*	67 (29.3)	267 (27.2)	6 (30.0)	17 (25.8)	128 (47.8)	341 (47.0)	11 (24.4)	41 (30.8)
*10–14*	138 (60.3)	614 (62.5)	4 (20.0)	18 (27.3)	121 (45.1)	305 (42.1)	12 (26.7)	27 (20.3)
*8–9*	14 (6.1)	72 (7.3)	2 (10.0)	6 (9.1)	9 (3.4)	51 (7.0)	6 (13.3)	11 (8.3)
*3–7*	10 (4.4)	30 (3.1)	8 (40.0)	25 (37.9)	10 (3.7)	28 (3.9)	16 (35.6)	54 (40.6)
Vomiting	8 (3.5)	21 (2.1)	1 (4.8)	3 (4.2)	15 (5.6)	38 (5.2)	6 (12.8)	14 (10.1)
Headache	6 (2.6)	19 (1.9)	–	4 (5.6)	12 (4.4)	16 (2.2)	-*	13 (9.4)
Anxiety	4 (1.7)	17 (1.7)	6 (28.6)	19 (26.8)	33 (12.2)	69 (9.4)	29 (61.7)*	58 (42.0)
Hallucinations	4 (1.7)	14 (1.4)	–	4 (5.6)	17 (6.3)	59 (8.0)	10 (21.3)	18 (13.0)
Agitation	28 (12.2)	102 (10.3)	6 (28.6)	29 (40.8)	74 (27.4)	209 (28.5)	17 (36.2)	54 (39.1)
Psychosis	3 (1.3)	17 (1.7)	1 (4.8)	2 (2.8)	23 (8.5)*	100 (13.6)	5 (10.6)	12 (8.7)
Seizures	3 (1.3)	10 (1.0)	–	6 (8.5)	5 (1.9)	13 (1.8)	8 (17.0)*	8 (5.8)
Cerebellar features	–	2 (0.2)	–	2 (2.8)	–	1 (0.1)	2 (4.3)	5 (3.6)
Palpitations	1 (0.4)	4 (0.4)	1 (4.8)	3 (4.2)	15 (5.6)	34 (4.6)	1 (2.1)	5 (3.6)
Chest pain	3 (1.3)	20 (2.0)	–	3 (4.2)	11 (4.1)	37 (5.0)	1 (2.1)	11 (8.0)
Arrhythmias	–	2 (0.2)	–	8 (11.3)	1 (0.4)	3 (0.4)	2 (4.3)	9 (6.5)
Lactate (mmol/L)^a,c,d^	–	–	0.8 (0.7–1.0)	1.1 (0.9–2.3)	–	–	1.7 (1.3–3.8)	2.0 (1.2–4.8)
Peak CK (IU/L)^c,e^	–	–	124 (80–175)**	366 (147–1270)	–	–	129 (78–285)*	233 (140–434)
Peak creatinine (mmol/L)^c,f^	–	–	58 (55–68)**	80 (59–105)	–	–	68 (61–74)***	82 (71–97)
Total	229 (100)	986 (100)	21 (100)	71 (100)	270 (100)	733 (100)	47 (100)	138 (100)

Percentages refer to total at the bottom of the column

^a^On presentation

^b^Missing: 6 in hospital opioid group (females: *n* = 20; males: *n* = 66), 7 in hospital non-opioid group (females: *n* = 45; males: *n* = 133)

^c^Median (interquartile range)

^d^Missing: 51 in hospital opioid group (females: *n* = 11; males: *n* = 30), 77 in hospital non-opioid group (females: *n* = 26; males: *n* = 82)

^e^Missing: 49 in hospital opioid group (females: n = 8; males: *n* = 35), 103 in hospital non-opioid group (females: *n* = 21; males: *n* = 61)

^f^Missing: 28 in hospital opioid group (females: n = 13; males: *n* = 51), 69 in hospital non-opioid group (females: *n* = 31; males: *n* = 85)

CK: creatine kinase; ED: emergency department; GCS: Glasgow Coma Scale; HR: heart rate per minute; RR: respiratory rate per minute; SBP: systolic blood pressure in mmHg; tp: temperature

*p < 0.05, **p < 0.01, ***p < 0.001 for comparisons between genders

Median age was lower among female cases in all groups (Table 2).

Gender differences in treatment, length of stay, and disposition were few (Table 2). However, a larger proportion of females in the outpatient opioid group required treatment beyond mere observation, 37.6% (86/229) vs 29.6% (292/986) (*p* = 0.024). Furthermore, fewer females were intubated, none (0/21) vs 21.1% (15/71) (*p* = 0.019) in the hospital opioid group, and 6.4% (3/47) vs 21.0% (29/138) (*p* = 0.039) in the hospital non-opioid group. Length of stay was shorter among females in the hospital opioid group, 14 h 56 min (IQR 4 h 17 min – 33 h 45 min) vs 21 h 25 min (IQR 11 h 14 min – 51 h 6 min) (*p* = 0.040).

Gender differences in clinical signs and symptoms were also few (Table 3). A larger proportion of females in the outpatient opioid group were hypotensive, 10.9% (25/229) vs 3.9% (38/986) (*p* < 0.001). A smaller proportion of females in the outpatient non-opioid group were psychotic, 8.5% (23/270) vs 13.6% (100/733) (*p* = 0.037). In the hospital non-opioid group, no females (0/47) reported headache vs 9.4% (13/138) (*p* = 0.041) among males, while larger proportions of females had seizures, 17.0% (8/47) vs 5.8% (8/138) (*p* = 0.031), and anxiety, 61.7% (29/47) vs 42.0% (58/138) (*p* = 0.030). Median peak CK and creatinine were lower among females in both hospital groups.

No patients died at the outpatient clinic. Two patients died in hospital, both were males having taken opioids.

## Discussion

### Summary of main findings

One in four cases of recreational drug toxicity were in females. The proportion of cases with GHB poisoning was larger in females both at the hospital and at the outpatient clinic, though absolute numbers were larger among males. There were few gender differences in the clinical course and treatment of acute recreational drug intoxication, even when comparing heavy drug users and party drug users separately.

### Gender differences

The small gender differences in our sample regarding clinical state and treatment are surprising, considering that female drug users report poorer health than males and have higher standardised mortality ratios [13, 15–17, 20]. We expected this to manifest itself in more severe clinical pictures when female drug users present to emergency medical services with acute recreational drug toxicity. Our results did not support this expectation. However, our patients were a heterogeneous group encompassing larger groups of party drug users than the more severely afflicted participants in most studies assessing gender differences in health among drug users [13–17, 20]. Still, when comparing only the patients taking opioids, as a proxy marker for heavy drug use, gender differences were few. We primarily expected the gender differences stemming from the poorer health among female drug users to appear as a more severe clinical course among the females in the opioid groups, but apart from hypotension, the few gender differences in clinical features were found in the non-opioid groups, hence not pertaining to the heavy drug users. It is also possible that the heavy drug users treated for overdose are a subgroup of heavy drug users, with even poorer health among both males and females. Hence, gender differences in health may be smaller in this marginalised group.

About one in four cases were in females, similar to previous studies of acute poisoning in Oslo in 2008 and 2012 [7, 8, 21], and similar to the gender differences in lifetime prevalence of drug use [1, 2]. Similar proportions of females and males were also found in a survey in seven Norwegian cities among people using illegal opioids and/or stimulants, 80% of whom injected drugs [2, 5]. Female patients were younger than males, in keeping with previous studies both on prevalence of use and on acute intoxications [2, 4, 5, 7, 21]. At both centres in our study, one in three GHB poisonings were in females, which is more than the one in four reported in the previous Oslo studies [7, 8, 21], and the one in six reported in a recent European multi-centre study [22]. One in five heroin poisonings were in females at the outpatient clinic and one in four at the hospital, similar to in the previous Oslo studies [7, 8, 21].

A larger proportion of females in the outpatient opioid group were hypotensive. Though hypotension is common among young women, this finding may reflect the possibility that females are more susceptible than males to the hypotensive effects of opioids. The higher peak creatinine and CK levels probably reflect normal physiological differences between genders rather than differences in toxic severity. From our findings, it seems that the clinical manifestations of acute recreational drug toxicity can be expected to be more or less the same in males and females. Patient management should be based on the clinical presentation, which currently is best practice in clinical toxicology.

A larger proportion of females in the outpatient opioid group required treatment beyond mere observation, though not due to antidote treatment with naloxone or flumazenil. No gender differences were found in the use of antidotes. Only three female patients were intubated at the hospital, compared to one in five males. Nearly all the intubated patients were also sedated, but we do not know which intervention was the primary one. However, there was no gender difference in agitation or psychosis among the hospital patients, nor in bradypnoea. Hence, we cannot explain the gender difference in intubation and sedation from the symptoms and signs recorded in our study. The larger proportion intubated and sedated may explain why males stayed longer in the hospital.

The study population was large. Together, the two locations treat a range of toxic severity similar to hospital emergency departments in other European countries. Hence, our findings are probably generalisable to emergency departments in countries with a similar spectrum of drug use. The larger proportions at the hospital of patients with symptoms and deranged vital signs requiring treatment, reflect the division of tasks between the outpatient clinic and the hospital: The more severely sick patients are treated at the hospital, and the vast majority of them are admitted to an intensive care unit.

### Limitations

Our study excluded poisoning with suicidal intention, which is more common among women than men, even when restricted to potential substances of abuse as the toxic agent [7, 8]. As mental health problems are more widespread among female drug users, excluding these poisonings might not give a fair comparison of the clinical state of women and men treated for acute poisoning in general. However, our aim in this study was specifically to compare the clinical state of patients with acute recreational drug toxicity between genders.

All data were collected from local medical records. There is most likely some variability in the recording between individual doctors and nurses, and we cannot rule out underreporting of clinical symptoms. This is especially the case for the most severely sick patients at the outpatient clinic, where rapid transfer to hospital often leads to short notes in the medical records. Accordingly, we might underestimate the severity of the intoxications at the outpatient clinic. Furthermore, diagnosis of toxic agents was mainly based on the assessment of the doctor treating the patient, not on toxicological testing. However, it is unlikely that either of these possible biases have any effect on the gender differences.

The variables collected may not be optimal to assess health in individuals, but they give an idea about the severity of a poisoning.

## Conclusions

One in four patients treated for acute recreational drug toxicity were women. Female patients were younger, and GHB was a more frequent toxic agent within the female group. Otherwise, the gender differences in clinical state and treatment were surprisingly small. Although female drug users report poorer health than males, this did not manifest itself in a more severe clinical course among women presenting with overdose.

## Abbreviations

CK: creatine kinase
ED: emergency department
GC-MS: gas chromatography – mass spectrometry
GCS: Glasgow Coma Scale
GHB: gamma-hydroxybutyrate
GP: general practitioner
HR: heart rate
LC-MS: liquid chromatography – mass spectrometry
LSD: lysergic acid diethylamide
MDMA: methylenedioxymethamphetamine
OAEOC: the Oslo Accident and Emergency Outpatient Clinic
OUH: Oslo University Hospital
RR: respiratory rate
SBP: systolic blood pressure
tp: temperature
